# Tuning the ferromagnetic phase in the CDW compound SmNiC_2_ via chemical alloying

**DOI:** 10.1038/srep26530

**Published:** 2016-05-25

**Authors:** G. Prathiba, I. Kim, S. Shin, J. Strychalska, T. Klimczuk, T. Park

**Affiliations:** 1 Department of Physics, Sungkyunkwan University, Suwon 440-746, Korea; 2 Faculty of Applied Physics and Mathematics, Gdansk University of Technology, Narutowicza 11/12, 80-233 Gdansk, Poland

## Abstract

We report a study on tuning the charge density wave (CDW) ferromagnet SmNiC_2_ to a weakly coupled superconductor by substituting La for Sm. X-ray diffraction measurements show that the doped compounds obey Vegard’s law, where La (Lu) alloying expands (shrinks) the lattice due to its larger (smaller) atomic size than Sm. In the series Sm_1−x_La_x_NiC_2_, CDW transition (*T*_CDW_ = 148 K) for SmNiC_2_ is gradually suppressed, while the ferromagnetic (FM) ordering temperature (*T*_C_) at 17 K slightly increases up to *x* = 0.3. For x > 0.3, *T*_C_ starts to decrease and there is no signature that could be related with the CDW phase. Electrical resistivity, magnetic susceptibility and specific heat measurements point toward the possible presence of a FM quantum critical point (QCP) near *x* = 0.92, where the *T*_C_ is extrapolated to zero temperature. Superconductivity in LaNiC_2_ (*T*_sc_ = 2.9 K) is completely suppressed with small amount of Sm inclusion near the proposed FM critical point, indicating a competition between the two ordered phases. The tunable lattice parameters via chemical substitution (La,Lu) and the ensuing change among the ordered phases of ferromagnetism, CDW and superconductivity underscores that SmNiC_2_ provides a rich avenue to study the rare example of a FM QCP, where the broken symmetries are intricately correlated.

Recently, study on a quantum critical point (QCP) has attracted great interest because of the possibility of previously unknown phases of matter from the singular quantum fluctuations associated with the QCP^1^. There exist a plethora of reports on antiferromagnetic (AFM) QCP systems, but examples with a ferromagnetic (FM) QCP are rare^2^,^3^,^4^. The CDW ferromagnet SmNiC_2_ shows a quasi-one-dimensional electronic structure, which leads to a CDW state from the Fermi surface nesting, e.g., Peierls instability^5^. Study on the effects of hydrostatic pressure in SmNiC_2_ has suggested that singular quantum fluctuations may create novel quantum phases in a vicinity of the projected FM quantum critical point (QCP) near 3.8 GPa (ref. 6). These novel phases, however, hide the presence of the QCP, requiring an alternative approach to probe the nature of the candidate FM QCP. Recently, Kim *et al*. predicted that both hydrostatic and chemical pressure have similar effects in tuning the electronic states of SmNiC_2_ (ref. 7).

Ternary rare-earth nickel carbides (RNiC_2_) were first reported by Bodak^8^, which are formed in an orthorhombic *Amm2* (space group #38) crystal structure with Ni and the rare-earth (R) metal chains being aligned along the crystallographic *a*-axis. This system is of particular interest because both CDW and long range magnetic ordering phases have been observed together. Among the RNiC_2_ compounds only two – SmNiC_2_ and LaNiC_2_ – exhibit properties other than an AFM ground state. Magnetism in RNiC_2_ originates from the lanthanide sublattice and displays an AFM ordering in most cases (R = Ce, Pr, Nd, Gd, Tb, Dy, Ho, Er, Tm)^9^,^10^. The exceptions, SmNiC_2_ and LaNiC_2_, are a ferromagnet and a superconductor, respectively. We note that the crystal structure of LuNiC_2_ has been reported^11^, but to our knowledge physical properties of this compound remain unknown.

In SmNiC_2_, the low-temperature synchrotron x-ray diffraction reveals that a CDW state with the wave vector **q** = (0.5, 0.52, 0) forms below 148 K and disappears below the Curie temperature 17.7 K, indicating destruction of the CDW due to appearance of the ferromagnetic ordering^12^. This is different from NdNiC_2_ where a CDW is still observed below the Nèel temperature (in the AFM state)^13^. The coexistence of a CDW with superconductivity is also observed in Er_5_Ir_4_Si_10_ and Lu_5_Ir_4_Si_10_ (refs 14 and 15).

Superconductivity in the first member of RNiC_2_ family, LaNiC_2_ with *T*_*sc*_ = 2.9 K, was first reported by W. H. Lee, *et al*. almost two decades ago^8^. Superconducting critical temperature can be increased to 8 K in the solid solution La_0.5_Th_0.5_NiC_2_ (ref. 16). In contrast, Y doping of La_1−x_Y_x_NiC_2_ decreases *T*_*sc*_ (ref. 17). Nuclear quadrupole relaxation (NQR)^18^ and specific heat^19^ measurements suggest that LaNiC_2_ is a conventional BCS superconductor. A pure singlet pairing state has been reported for the non-centrosymmetric superconductors: Li_2_Pd_3_B (ref. 20), BaPtSi_3_ (ref. 21), Re_3_W (ref. 22), Mg_10_Ir_19_B_16_ (ref. 23). However, strong evidences for an unconventional character of superconductivity in LaNiC_2_ have been recently suggested by muon spin relaxation (μSR)^24^,^25^ and penetration depth experiments^26^. In addition, a phenomenological two-gap BCS model was recently proposed by Kim *et al*.^7^. The uncertainty on the nature of superconductivity for LaNiC_2_ makes it more important to study the relationship between superconductivity and ferromagnetism.

Here we report crystallographic and physical properties of Sm_1−x_La_x_NiC_2_ and Sm_1−y_Lu_y_NiC_2_, where La and Lu were substituted for Sm to introduce negative and positive chemical pressures, respectively. X-ray diffraction study showed that the volume change in the La (or Lu) alloyed compounds obeys Vegards’ law: La (Lu) alloying expands (shrinks) the volume linearly. Since La is an element with empty 4*f* orbital and Lu has a fully filled 4*f* orbital with *J* = 0, the chemical alloying does not introduce magnetism to the system, but is expected to induce chemical pressure effects through a change in the distance between constituent elements. Magnetic susceptibility, electrical resistivity and heat capacity data point toward the possible presence of a FM quantum critical point near *x* = 0.92, where the Curie temperature *T*_*C*_ is extrapolated to zero temperature and the specific heat divided by temperature is strongly enhanced with decreasing temperature down to the lowest measuring temperature. Comprehensive magnetic and superconducting phase diagram for both Sm_1−x_La_y_NiC_2_ and Sm_1−y_Lu_y_NiC_2_ is constructed for the first time.

## Results

### X-ray Diffraction Measurements

**Figure 1:**
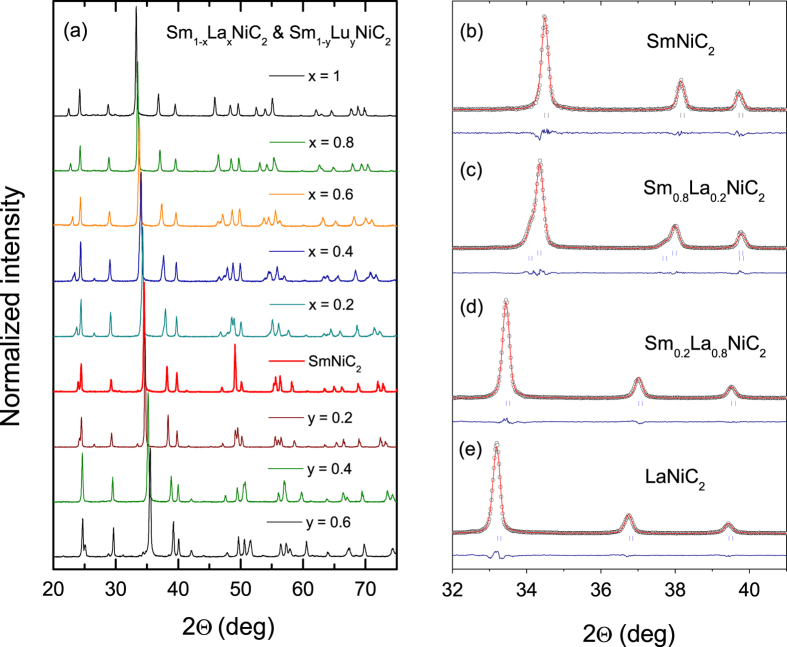
Powder x-ray diffraction (XRD) patterns. (**a**) XRD for the series Sm_1−x_La_x_NiC_2_ for *x* = 0, 0.2, 0.4, 0.6, 0.8, 1 and Sm_1−y_Lu_y_NiC_2_ for y = 0.2, 0.4, 0.6. (**b**) Enlarged image of the main peak (111) showing a shift from right to left for SmNiC_2_ and LaNiC_2_ respectively. Open circles are experimental points, whereas calculated diffraction patterns is represented by a solid red line. Difference between experiment and a model is shown by a blue line. The vertical ticks correspond to the Bragg peaks for Sm_1−x_La_x_NiC_2_.

Powder XRD patterns are presented in Fig. 1(a) for Sm_1−x_La_x_NiC_2_ and Sm_1−y_Lu_y_NiC_2_. Main reflection peaks clearly shift towards lower and higher angles for Sm_1−x_La_x_NiC_2_ and Sm_1−y_Lu_y_NiC_2_, respectively. This is consistent with the larger (smaller) ionic radius of La^3+^ (Lu^3+^) than Sm^3+^ and confirms successful chemical alloying. The panels (b–e) of Fig. 1 present the Le Bail refinements performed using the FULLPROF diffraction suite^27^ for representative Sm_1−x_La_x_NiC_2_ (x = 0, 0.2, 0.8 and 1.0) samples. The refinements confirm an orthorhombic *Amm2* crystal structure (s.g. #38) and give the lattice parameters for Sm_1−x_R_x_NiC_2_ (R = La and Lu). More detailed inspection of the Sm_1−x_La_x_NiC_2_ series of the XRD patterns reveals a slight broadening and splitting in the main reflection peaks for 0.2 ≤ x ≤ 0.6. An excellent Le Bail fit (R_wp_ = 11.7, R_exp_ = 8.65, *χ*^2^ = 1.84) was obtained by assuming the presence of two phases with the same crystal structure and different lattice parameters as can be seen for Sm_0.8_La_0.2_NiC_2_ (see Fig. 1c). This suggests that two distinct compounds Sm_1−x_La_x_NiC_2_ with slightly different La concentrations (*x*) are present for the intermediate composition range. The majority phase is the one with smaller *x* – the Sm-richer variant. Such behaviour is not observed for Lu-doped series. The refined lattice parameters for Sm_1−x_La_x_NiC_2_ are shown in Fig. S1 of the Supplementary Information. The *a* and *c* lattice parameters obey Vegard’s law for the whole La concentration range, while the *b* parameter is almost constant up to x = 0.3 and linearly increases with further increasing La level. The rigid C-C dimers along the *b* direction are likely responsible for the negligible change in the *b* lattice constant for the lower La level^10^. The intermediate region, in which two phases with different La/Sm ratio are present, is shadowed on the diagram and the lattice parameters for the second, minority phase are shown by triangles.

**Figure 2:**
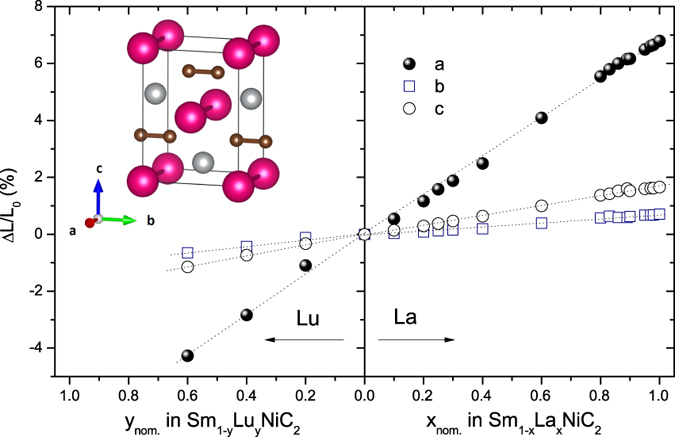
Relative change in lattice parameters: ΔL/L_0_ = (L_x_−L_0_)/L_0_ and ΔL/L_0_ = (L_y_−L_0_)/L_0_ for La and Lu doped samples, respectively. The lattice parameter along the *a*-axis (solid circles) shows the maximal change. Inset shows the crystal structure of SmNiC_2_. Samarium and nickel atoms are represented by purple and grey balls, respectively. Small carbon atoms are marked by brown balls. Note that the C-C dimers are located along the *b* – axis.

The relative change (ΔL/L_0_) of each lattice parameter vs. nominal concentration of La and Lu for Sm_1−x_La_x_NiC_2_ and Sm_1−y_Lu_y_NiC_2_ is presented in Fig. 2. ΔL is the change of each lattice parameter compared to the lattice parameter (L_0_) of the parent (SmNiC_2_) compound. For example Δ*a*/*a*_0_ was calculated from (*a*_x_ − *a*_0_)/*a*_0_, where *a*_x_ and *a*_0_ are the *a*-axis lattice parameters for Sm_1−x_La_x_NiC_2_ and SmNiC_2_, respectively. With increase in *x*, the lattice parameter along the *a*-axis expands more rapidly than that along the *b-* and *c-*axis: as large as a 7% increase in the *a*-axis is observed, while less than 1% change occurs along the b-axis. In contrast, Lutetium doping (*y*) causes a decrease of the lattice parameters, which is most pronounced along the *a-*axis.

### Electrical Resistivity

**Figure 3:**
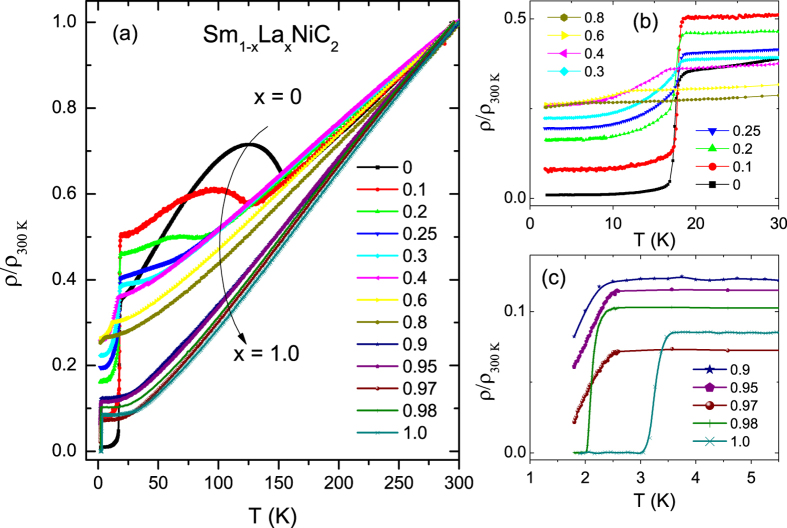
Electrical resistivity for La-doped SmNiC_2_. (**a**) Temperature dependence of the normalized electrical resistivity for Sm_1−x_La_x_NiC_2_, 0 ≤ x ≤ 1. Low-temperature resistivity plots of the ferromagnetic (0 ≤ x ≤ 0.8) and superconducting transitions (0.9 ≤ x ≤ 1) are shown in (**b**,**c**), respectively.

Resistivity measurements were performed for Sm_1−x_La_x_NiC_2_ (0 ≤ x ≤ 1) and Sm_1−y_Lu_y_NiC_2_ (0 ≤ y ≤ 0.4) series. Temperature dependence of the normalized resistivity is shown in Fig. 3 for Sm_1−x_La_x_NiC_2_, where resistivity values are normalized to those at 300 K for comparison. Depending on the La concentration, three features are observed. For the parent SmNiC_2_ and slightly La doped (x < 0.3) Sm_1−x_La_x_NiC_2_, a sharp inflection at high temperature is seen due to a charge density wave (CDW) formation. *T*_CDW_ was assigned as the minimum of the temperature derivative of resistivity (d*ρ*/d*T*). For the parent compound SmNiC_2_ the obtained *T*_CDW_ (=148 K) is comparable with the previous reports^10^,^28^. With increase in La content, the CDW transition temperature starts to decrease rapidly, reaches to 34 K for Sm_0.7_La_0.3_NiC_2_, and is not observed for *x* > 0.3. The increase in resistivity just below *T*_CDW_ is due to a CDW gap opening on the Fermi surface. For SmNiC_2_ resistivity reaches a maximum at about 120 K and decreases with further decrease of temperature. Another feature that is clearly visible for SmNiC_2_ is a sharp drop in resistivity at 17.2 K (see Fig. 3(b)), which is caused by the ferromagnetic (FM) phase transition, as will be supported by magnetic measurements. Although such behaviour is typically seen for ferromagnetic compounds below the Curie temperature, one order of magnitude decrease of resistivity is rare and may originate from the destruction of the CDW state at the same temperature. When a CDW is present, ferromagnetism is robust. In fact, there is a slight increase in the Curie temperature from 17.2 K (SmNiC_2_) to 17.8 K (Sm_0.25_La_0.75_NiC_2_) with increasing *x*. For *x* > 0.3, the CDW transition is no longer observed and the Curie temperature starts to decrease with increasing La concentration, which suggests a strong correlation between the CDW and ferromagnetic phases.

Figure 3(c) presents resistivity data for Sm_1−x_La_x_NiC_2_ system with high La concentration (*x* ≥ 0.9). A sharp superconductivity transition is observed for LaNiC_2_ and Sm_0.02_La_0.98_NiC_2_ with *T*_sc_ = 3.4 K and 2 K, respectively. The samples with slightly lower *x* (0.97 and 0.95) do not show a transition to the zero-resistance superconducting state above 1.8 K, suggesting that the resistivity drop is due to filamentary nature of the SC phase. The rapid suppression of superconductivity is often observed in the presence of magnetic impurities that act as strong scattering centers to destroy superconducting Cooper pairs. One such example is La_1−x_Gd_x_ alloy, where only 1% of Gd alloying suppresses *T*_sc_ from almost 6 K for La to 1 K for La_0.99_Gd_0.01_ (ref. 29).

In contrary to La alloying, the low-*T* normalized resistivity of SmNiC_2_ is enhanced by Lu alloying (see Fig. S2 in the Supplementary Information). The slope of the resistivity (d*ρ*/d*T*) at high temperatures decreases with increase of Lu concentration, too. The 20% Lu-doped compound (Sm_0.8_Lu_0.2_NiC_2_) reveals both CDW and FM behaviour. The Curie temperature is suppressed to 10 K, whereas the CDW transition temperature increases to 152 K, a 4 K increase from the pure compound. It is in contrast to the La-doping (Sm_1−x_La_x_NiC_2_): at the same concentration level (x = 0.2), *T*_CDW_ is suppressed to 126 K, a 24 K decrease.

### Magnetization Measurement

**Figure 4:**
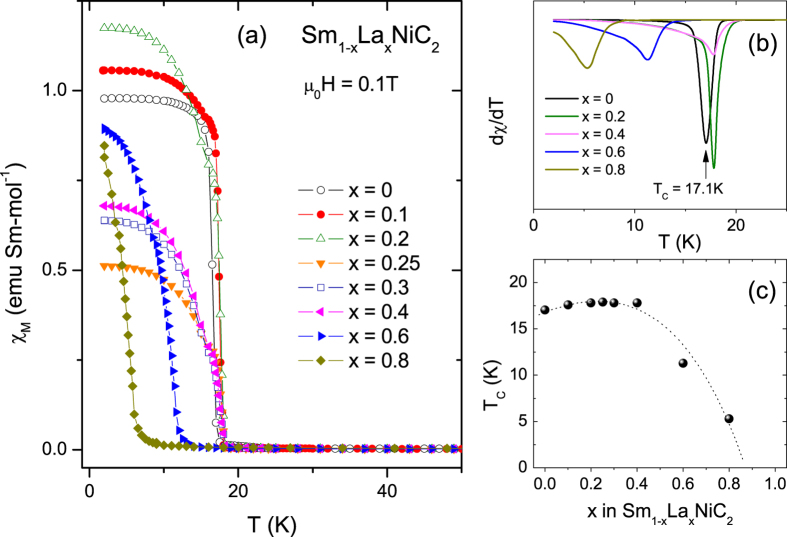
Magnetization for La-doped SmNiC_2_. (**a**) Molar magnetic susceptibility *χ*_M_ plotted against temperature for Sm_1−x_La_x_NiC_2_, 0 ≤ x ≤ 0.80. (**b**) First temperature derivative of the susceptibility is representatively shown to determine the FM transition temperature *T*_C_ for *x* = 0, 0.2, 0.4, 0.6, 0.8. Arrow marks the *T*_C_ for *x* = 0, where d*χ*_M_/d*T* is a minimum. (**c**) Evolution of *T*_C_ as a function of La concentration *x*.

**Figure 5:**
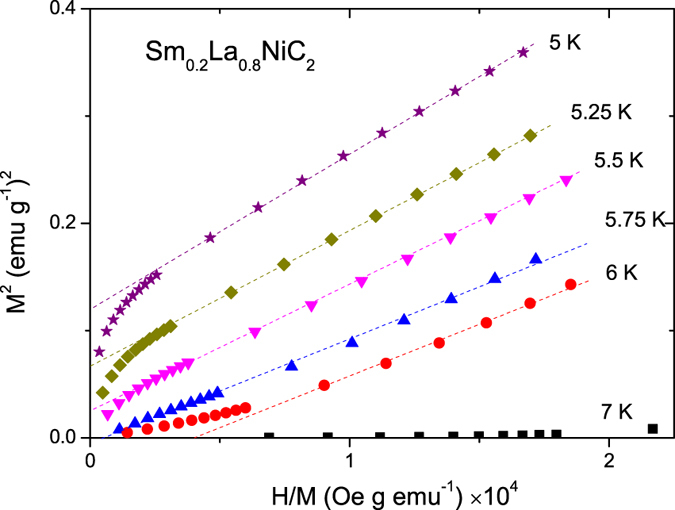
Arrott plot for Sm_1−x_La_x_NiC_2_ with *x* = 0.8. Square of the isothermal magnetization *M*^2^ is plotted against *H*/*M* for several temperatures between 5 K and 7 K. Dashed lines are guides to the eyes.

Magnetic characterization of the Sm_1−x_La_x_NiC_2_ series is presented in Fig. 4. There are no anomalies at around 13 K and 25 K that originates from the presence of SmNi_2_ binary phase^9^. The absence of anomalous features suggests high quality of our samples. The molar magnetic susceptibility (*χ*_M_) at *μ*_*o*_*H* = 0.1 T (Fig. 4a) shows a rapid increase with decreasing temperature below 17 K and saturates between 0.5 to 1.2 emu/Sm mol for different La concentrations at low temperatures. At this moment, it is difficult to point out a correlation between the saturated moments and alloying concentrations because the samples are in a polycrystalline form. The FM transition temperature determined by the minimum of the temperature derivative of susceptibility (d*χ*/dT), as shown in Fig. 4b, is in good agreement with the Curie temperature *T*_C_ = 17.2 K from the resistivity measurement. Ferromagnetism in Sm_1−x_La_x_NiC_2_ is robust. *T*_C_ initially increases with La concentration, reaches a maximum of 18 K at *x* = 0.25, and then decreases with further increasing La level, showing a small dome shape in the *T-x* phase diagram. It is interesting to note that the ferromagnetic phase persists up to the nominal La concentration *x* = 0.86: a ferromagnetic transition at as low as 2.5 K was observed for the composition *x* = 0.86 in the series Sm_1−x_La_x_NiC_2_. In order to confirm the ferromagnetic state and precisely estimate the Curie temperature, the Arrott plot analysis was performed. A series of isothermal magnetisation curves in the immediate vicinity of the Curie temperature were measured. In a plot of *H*/*M* vs *M*^2^, the isotherm which passes through the origin gives the best estimate of Curie temperature because *H*/*M* = *βM*^*2*^ at *T* = *T*_C_ (ref. 30). The Arrott plot presented in Fig. 5 shows that the ferromagnetic transition temperature for Sm_0.2_La_0.8_NiC_2_ is 5.5 K, very close to *T*_C_ = 5.3 K estimated for the same sample from the minimum of d*χ*/d*T*. Using the Arrott plot, *T*_C_ = 3.5 K was estimated for Sm_0.14_La_0.86_NiC_2_ (not shown here).

A molar magnetic susceptibility at 300 K for the Sm_1−x_La_x_NiC_2_ series is shown in Fig. S3 of the Supplementary Information, which linearly decreases with increasing La concentration. As expected, the extrapolated line reaches zero at LaNiC_2_. The same experimental data normalized per Sm-mol is almost independent of *x* and is about *χ*(300K) = 1.8 × 10^−3 ^emu/Sm-mol, indicating that La is successfully substituted for Sm.

According to H. Onodera *et al*., spontaneous magnetization (*M*) along the *a*-axis of the single crystal (~0.32 μ_B_) is smaller than 0.72 μ_B_ for Sm^3+^, while it is negligible along the *b*- and *c*-axis^9^. The fact that we have obtained 0.19 μ_B_ at 2 K could be ascribed to the polycrystalline form of the measured sample. Such a small value has been explained by the mixed valence state of Sm^2+^ and Sm^3+^ ions or crystalline electric field (CEF) effects. A mixed valent state is common in Sm containing compounds. Above the ferromagnetic transition temperature the entire series did not follow the Curie-Weiss behaviour, which might be pertinent to the mixed valent state of Sm ions. It is imperative to study the exact nature of magnetic interactions present in SmNiC_2_ by measurements like neutron diffraction to gain a deep insight on the valence of Sm ions.

### Heat Capacity Measurements

**Figure 6:**
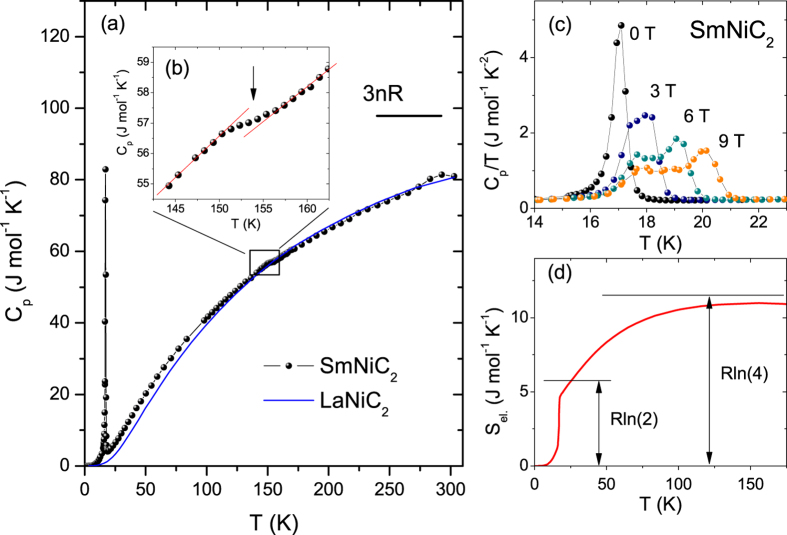
Specific heat of La-doped SmNiC_2_. (**a**) Temperature dependence of the specific heat *C*_p_ for SmNiC_2_ (black dots) and LaNiC_2_ (blue line). The inset (**b**,**c**) show *C*_p_ in the vicinity of the CDW and PM-FM transition, respectively. The inset (**d**) displays the magnetic entropy as a function of temperature for SmNiC_2_.

Heat capacity *C*_p_(*T*) of the polycrystalline SmNiC_2_ (black dots) and LaNiC_2_ (blue solid line) is plotted as a function of temperature in Fig. 6(a). At the highest temperature, *C*_p_ (at 300 K) reaches approximately 80% of the value expected by Dulong-Petit law *3nR* value ~100 J mol^−1^K^−1^, suggesting that the Debye temperature for SmNiC_2_ exceeds 300 K. A small anomaly at around 153K (inset b) is likely caused by the CDW ordering, although this temperature is 5 K higher than a CDW temperature estimated from the resistivity and magnetization measurements. A huge anomaly is visible at low temperature and details are presented in Fig. 6(c). In the zero-field data, a λ-shape transition occurs at *T*_C_ = 17 K, which is in agreement with the Curie temperature estimated by resistivity and magnetization techniques. With increasing magnetic field, the transition is broadened and split into two peaks, indicating an additional field-induced phase transition. A simple subtraction of the LaNiC_2_ specific heat from the SmNiC_2_ specific heat yields the temperature dependence of the magnetic specific heat (not shown). The integrated entropy, presented in Fig. 6(d), is close to *R*ln4 at about 150 K, comparable to the CDW transition temperature for SmNiC_2_. The magnetic entropy recovered at *T*_C_ ( = 17.1 K) accounts for 80% of *R*ln2, the entropy expected for the doublet ground state of the *J* = 5/2 multiplet for Sm^3+^ (4f 6). Incomplete recovery of the entropy at *T*_*C*_ could be ascribed to either the fluctuating valence between Sm^3+^ and Sm^2+^ or the Kondo screening effects of Sm 4*f* spins by the itinerant electrons.

**Figure 7:**
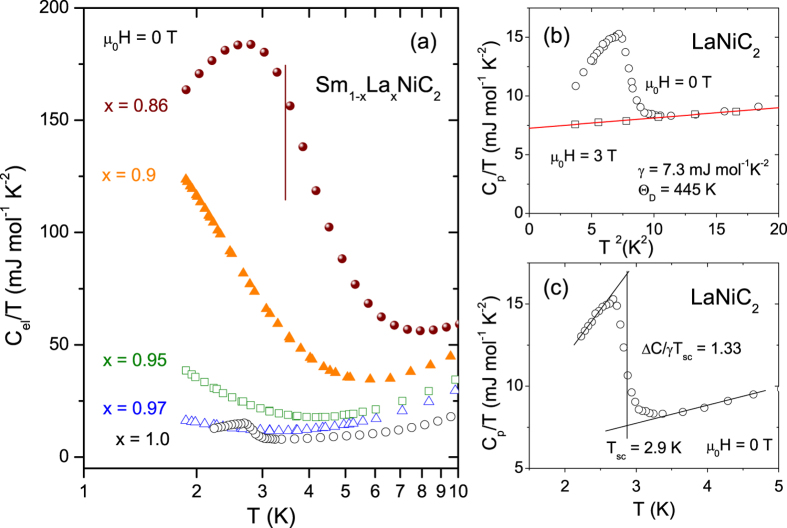
Low temperature specific heat data of Sm_1−x_La_x_NiC_2_ (0.86 < x < 1.0). The vertical line mark the Curie temperature estimated from the Arrott plot of the magnetization. Panel (**b**) shows *C*_p_/*T* versus *T* ^2^ for LaNiC_2_ under zero magnetic field (open circles) and 3T (open squares). The solid red line represents fit to *C*_*p*_/*T* = γ + β*T*^*2*^. Panel (**c**) shows *C*_p_/*T* versus *T*_sc_ in the vicinity of superconducting transition. Solid line shows the equal entropy construction.

Heat capacity data (*C*_*p*_/*T*) for La-rich samples is selectively presented on a semi-logarithmic scale in Fig. 7. A sharp SC transition for LaNiC_2_ (open circles) is visible at 2.9 K in Fig. 7(a). When Sm is alloyed in LaNiC_2_, the SC jump in the specific heat is quickly suppressed and there is no signature for the SC transition down to 1.8 K, the lowest measured temperature, for 3% Sm concentration (x = 0.97). With further increasing Sm concentration, the low-temperature specific heat divided by temperature (*C*/*T*) increases with decreasing temperature and shows a peak at 14% Sm concentration due to the FM ordering, where the brown vertical line marks the Curie temperature estimated from the Arrott plot of magnetization. Even though the lowest measured temperature is limited to 1.8 K, the singular enhancement in *C*/*T* is clearly visible as Sm concentration approaches 10%, indicating the possible presence of a FM quantum critical point at that concentration.

Figure 7(b,c) magnifies the specific heat of LaNiC_2_ near the SC transition temperature. The normal-state specific heat measured at μ_0_*H* = 3 T, shown by open squares in Fig. 7b, was fitted to *C*_p_ = γ*T* + β*T* ^3^, where the first and second terms represent electronic and lattice contributions, respectively. The fit to these data allows us to estimate γ = 7.3(1) mJ mol^−1 ^K^−2^, and β = 0.088(5) mJ mol^−1 ^K^−4^. The simple Debye model connects the β coefficient and the Debye temperature *Θ*_D_ through *Θ*_*D*_ = (*12π*^*4*^*nR*/*5*β)^*1*/*3*^ = 445 K, where R = 8.314 J mol^−1 ^K^−1^ and *n* is the number of atoms per formula unit (*n* = 4 for LaNiC_2_). This value is slightly lower than reported *Θ*_D_ = 456 K for YNiC_2_ (ref. 31) and can be explained by a simple mass relationship: larger La mass than Y should result in lower *Θ*_D_.

From an equal entropy construction shown by the solid lines in Fig. 7c, superconducting critical temperature was obtained to be *T*_sc_ = 2.9 K, which is lower than that from the resistivity measurement (*T*_sc_ = 3.4 K). The ratio between the specific heat jump (Δ*C*) at *T*_c_ and the Sommerfeld coefficient (γ), Δ*C*/γ*T*_sc_, is 1.33, which is close to the BCS predicted value of 1.42 for a weakly coupled superconductor.

## Discussion

**Figure 8:**
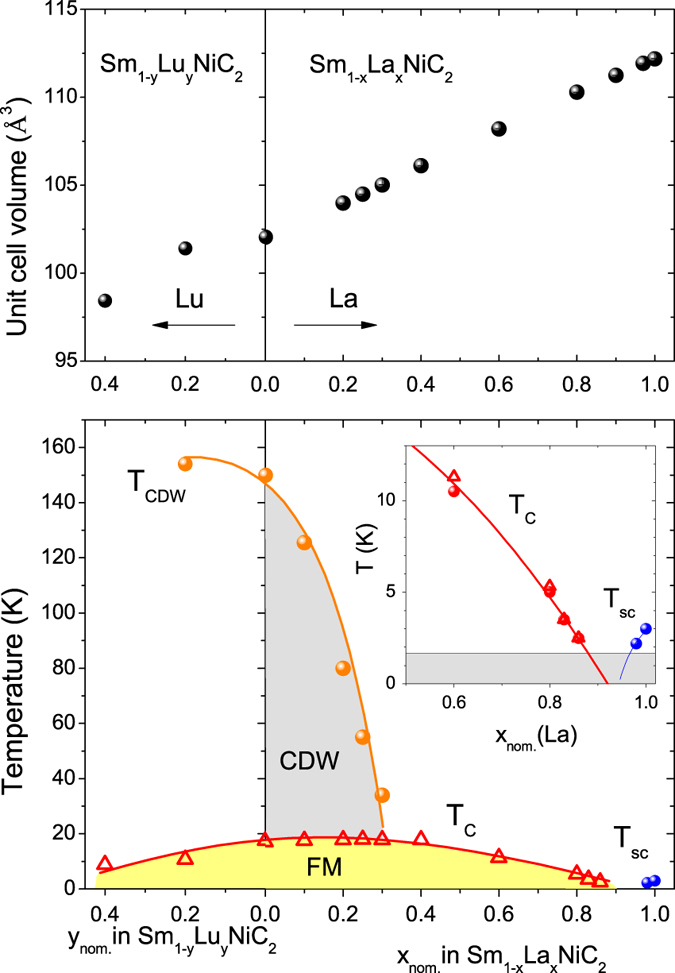
Temperature vs concentration phase diagram. Top panel shows the change in volume of the orthorhombic series Sm_1−x_La_x_NiC_2_ (0 ≤ *x* ≤ 1) and Sm_1−y_Lu_y_NiC_2_ (0 ≤ *y* ≤ 0.4) with respect to composition. The bottom panel shows the phase diagram of temperature vs. composition. For Sm_1−x_La_x_NiC_2_, the CDW ordering disappears above *x* = 0.3, while the ferromagnetic phase survives up to *x* = 0.86. The superconductivity is rapidly suppressed for *x* < 1 in Sm_1−x_La_x_NiC_2_. For the Lu-doped Sm_1−y_Lu_y_NiC_2_, T_CDW_ disappears for *y* > 0.2 and the magnetic phase decreases with increasing Lu concentration *y*. The open triangles are data points obtained from the magnetization measurements, while the solid circles are from resistivity measurements.

It is interesting to note that the CDW phase is driven by the one-dimensional (1D) anisotropy along the *a*-axis in the electronic structure^7^. The 1D anisotropy increases with chemical or physical pressure mainly because the Ni (or Sm) chain along the *a*-axis is affected the most (either compressed or expanded). The phase diagram for the series Sm_1−x_La_x_NiC_2_ is shown in Fig. 8. The results from both transport and magnetic measurements are used to plot the phase diagram. In the top panel of Fig. 8, the unit cell volume is plotted against *x* and *y*, which evidently shows that both Sm_1−x_La_x_NiC_2_ and Sm_1−y_Lu_y_NiC_2_ follow Vegard’s law. In the bottom plot of Fig. 8, we can see a slight increase in *T*_CDW_ with the inclusion of Lu in the lattice due to a positive chemical pressure, while there occurs a decrease in *T*_CDW_ with increase in La due to a negative chemical pressure in the lattice. The CDW transition is getting suppressed from 148 K (*x* = 0) to 34 K (*x* = 0.3) with La doping concentration *x* and completely destroyed for x > 0.3.

When a CDW is present, there is a slight increase in the FM Curie temperature from 17.2 K to 17.8 K (which is confirmed from both transport and magnetic measurements). Recently B-doped SmNiC_2_ reported a similar increase in *T*_C_ from 17.5 K to 23.1 K until the CDW is present^32^. Once a CDW is suppressed in Sm_1−x_La_x_NiC_2_, however, the Curie temperature starts to decrease and is suppressed down to 2.5 K at *x* = 0.86. Inset of the bottom panel magnifies the temperature-La concentration phase diagram near the pure LaNiC_2_, where solid lines are guides to eyes. Both *T*_C_ and *T*_sc_ could be extrapolated to zero Kelvin at x = 0.92, underscoring the possibility of a FM QCP that was proposed by the singular enhancement in the low-*T* specific heat. Electrical resistivity measurements show that the first-order FM transition in SmNiC_2_ changes to the second order or a weakly first order for higher La concentration, suggesting that the disorder introduced by La substitution may be important to the realization of the FM QCP in SmNiC_2_ (see Fig. S4 in the Supplementary Information). In the case of Lu doped SmNiC_2_, *T*_C_ is strongly suppressed from 17.5 K to 8.8 K for *y* = 0.4. As shown in Fig. S2 in the Supplementary Information, however, the disparate resistivity behaviour near *T*_C_ for *y* = 0.4 demands further investigation on the precise nature of magnetic ordering for different Lu doped level.

FM ordering persists up to x = 0.92 in LaNiC_2_-SmNiC_2_ system, where the dilution of Sm local moments by La substitution exceeds the percolation limit^33^. Once a CDW is destroyed, the relationship in LaNiC_2_-SmNiC_2_ solid solution is almost similar to La-Gd alloy system, where the SC transition temperature of La elemental metal decreases rapidly with increasing Gd content and is completely suppressed at 0.9 at% Gd. La-Gd alloy containing just 3% Gd becomes ferromagnetic at 1.3 K (ref. 29). However, Gd in Y did not show any FM until 10 at% Gd^34^. With the introduction of other rare earths in binary alloys the Neel and Curie points are generally lowered. We have also synthesized other rare earth substitution such as Y alloying to SmNiC_2_. For 20% Y concentration, the CDW is completely destroyed and the Curie temperature is also dropped to 10 K (not shown here).

## Conclusions

We have successfully synthesized SmNiC_2_ solid solution with La (or Lu) and investigated the tuneable behaviour from the CDW ferromagnet to the weakly coupled superconductor. La alloying in SmNiC_2_ expands the lattice parameters (negative pressure), while Lu alloying shrinks the lattice parameters (positive pressure). The CDW transition temperature (*T*_CDW_ = 148 K) in Sm_1−x_La_x_NiC_2_ decreases with increasing La inclusion *x* because of the poor Fermi surface nesting conditions from the La alloying. La (or Lu) alloying also dilutes the density of Sm local moments because there is no f electron in the substituent, therefore suppressing the FM phase. The Curie temperature, however, does not decrease monotonically with La concentration, but shows a maximum near x = 0.3, underscoring that the CDW and ferromagnetic phases compete against each other. Superconductivity is observed only for La rich compounds (x > 0.92), where the SC transition temperature (*T*_sc_ = 3.4 K) for LaNiC_2_ is quickly suppressed with increasing Sm contents and to zero Kelvin near 8% Sm concentration. When combined with the fact that both Curie temperature and SC transition temperature is suppressed to zero Kelvin near *x*_c_ = 0.92, the singular enhancement of the low-*T* specific heat at the critical concentration *x*_c_ points toward the presence of a ferromagnetic quantum critical point (QCP). We note that disorder introduced by the La substitution may be conducive to the realization of the FM QCP in SmNiC_2_ (ref. 35). More study is in progress to elucidate the nature of the candidate QCP.

## Methods

The series of compounds Sm_1−x_La_x_NiC_2_ (0 ≤ x ≤ 1) and Sm_1−y_Lu_y_NiC_2_ (0 ≤ y ≤ 0.4) were synthesized by the arc-melting technique, using constituent elements of purity 99.9% or higher. The weight loss after arc melting was less than 1%, indicating that the nominal concentration is close to the actual alloying level. Since WDS analysis corroborates this conclusion, the nominal concentration was used throughout this manuscript. The arc-melted samples were annealed at 1173K for ten days in a sealed evacuated quartz tube. The annealed samples were quenched in NaCl-ice water mixture. Structural characterization was performed by the powder x-ray diffraction (PXRD) method using a Rigaku diffractometer with Cu Kα radiation. The lattice parameters of the samples were determined by LeBail profile refinements of PXRD carried out using the FULLPROF software^27^. Resistivity measurements were performed using a standard four probe technique employing a Quantum Design Physical Property Measurement System (PPMS). The contacts were made by spot welding of platinum wires on the sample surface. Heat capacity was measured in temperature range 1.9 K < T < 300 K at fields up to 9 T by using the thermal relaxation technique (PPMS system). Magnetic measurements were carried out using a Quantum Design Magnetic Property Measurement System (MPMS).

## Additional Information

**How to cite this article**: Prathiba, G. *et al*. Tuning the ferromagnetic phase in the CDW compound SmNiC_2_ via chemical alloying. *Sci. Rep.* **6**, 26530; doi: 10.1038/srep26530 (2016).

## Supplementary Material

Supplementary Information

## References

[b1] Coleman P. & Schofield A. J. Quantum criticality. Nature 433, 226–229 (2005). 15662409 10.1038/nature03279

[b2] Lohneysen H., Rosch A., Vojta M. & Wolfle P. Fermi-liquid instabilities at magnetic quantum phase transitions. Rev. Mod. Phys. 79, 1015–1075 (2007).

[b3] Stewart G. R. Non-Fermi-liquid behaviour in d- and f-electron metals. Rev. Mod. Phys. 73, 797–855 (2001).

[b4] Steppke A. . Ferromagnetic Quantum Critical Point in the Heavy-Fermion Metal YbNi_4_(P_1−x_As_x_)_2_. Science 339, 933–936 (2013). 23430650 10.1126/science.1230583

[b5] Zhu X., Cao Y., Zhang J., Plummer E. W. & Guo J. Classification of charge density waves based on their nature. Proc. Natl. Acad. Sci. 112, 2367–2371 (2015). 25646420 10.1073/pnas.1424791112 PMC4345626

[b6] Woo B. . Effects of pressure on the ferromagnetic state of the charge density wave compound SmNiC_2_. Phys. Rev. B 87, 125121 (2013).

[b7] Kim J. N., Lee C. & Shim J.-H. Chemical and hydrostatic pressure effect on charge density waves of SmNiC_2_. New J. Phys. 15, 123018 (2013).

[b8] Bodak O. I. & Marusin E. P. Crystal structure of CeNiC_2_, LaNiC_2_, PrNiC_2_ compounds. *Dopov*. Akad. Nauk Ukr. SSR 12, 1048–1050 (1979).

[b9] Onodera H. . Magnetic properties of single-crystalline RNiC_2_ compounds (R = Ce, Pr, Nd and Sm). J. Magn. Magn. Mater. 182, 161–171 (1998).

[b10] Murase M. . Lattice Constants, Electrical Resistivity and Specific Heat of RNiC_2_. J. Phys. Soc. Jpn. 73, 2790–2794 (2004).

[b11] Jeitschko W. & Gerss M. H. Ternary carbides of the rare earth and iron group metals with CeCoC_2_- and CeNiC_2_-type structure. J. Common Met. 116, 147–157 (1986).

[b12] Shimomura S. . Charge-Density-Wave Destruction and Ferromagnetic Order in SmNiC_2_. Phys. Rev. Lett. 102, 076404 (2009). 19257698 10.1103/PhysRevLett.102.076404

[b13] Yamamoto N., Kondo R., Maeda H. & Nogami Y. Interplay of Charge-Density Wave and Magnetic Order in Ternary Rare-Earth Nickel Carbides, RNiC_2_ (R = Pr and Nd). J. Phys. Soc. Jpn. 82, 123701 (2013).

[b14] Galli F. . Coexistence of charge density wave and antiferromagnetism in Er_5_Ir_4_Si_10_. J. Phys. Condens. Matter 14, 5067 (2002).

[b15] Becker B. . Strongly coupled charge-density wave transition in single-crystal Lu_5_Ir_4_Si_10_. Phys. Rev. B 59, 7266–7269 (1999).

[b16] Lee W. H. & Zeng H. K. Superconductivity in the series (La_1−x_Th_x_)NiC_2_ (0 ≤ x ≤ 0.8). Solid State Commun. 101, 323–326 (1997).

[b17] Liao T. F., Sung H. H., Syu K. J. & Lee W. H. Alloying effects of Y on in superconducting (La_1−x_Y_x_)NiC_2_. Solid State Commun. 149, 448–452 (2009).

[b18] Iwamoto Y., Iwasaki Y., Ueda K. & Kohara T. Microscopic measurements in 139La-NQR of the ternary carbide superconductor LaNiC_2_. Phys. Lett. A 250, 439–442 (1998).

[b19] Pecharsky V. K., Miller L. L. & Gschneidner K. A. Low-temperature behavior of two ternary lanthanide nickel carbides: Superconducting LaNiC_2_ and magnetic CeNiC_2_. Phys. Rev. B 58, 497–502 (1998).

[b20] Yuan H. Q. . S-Wave Spin-Triplet Order in Superconductors without Inversion Symmetry: Li_2_Pd_3_B and Li_2_Pt_3_B. Phys. Rev. Lett. 97, 017006 (2006). 16907402 10.1103/PhysRevLett.97.017006

[b21] Bauer E. . BaPtSi_3_: A noncentrosymmetric BCS-like superconductor. Phys. Rev. B 80, 064504 (2009).

[b22] Zuev Y. L. . Evidence for s-wave superconductivity in noncentrosymmetric Re_3_W from magnetic penetration depth measurements. Phys. Rev. B 76, 132508 (2007).

[b23] Aczel A. A. . Muon spin rotation/relaxation measurements of the noncentrosymmetric superconductor Mg_10_Ir_19_B_16_. Phys. Rev. B 82, 024520 (2010).

[b24] Quintanilla J., Hillier A. D., Annett J. F. & Cywinski R. Relativistic analysis of the pairing symmetry of the noncentrosymmetric superconductor LaNiC_2_. Phys. Rev. B 82, 174511 (2010).

[b25] Hillier A. D., Quintanilla J. & Cywinski R. Evidence for Time-Reversal Symmetry Breaking in the Noncentrosymmetric Superconductor LaNiC_2_. Phys. Rev. Lett. 102, 117007 (2009). 19392234 10.1103/PhysRevLett.102.117007

[b26] Bonalde I., Ribeiro R. L., Syu K. J., Sung H. H. & Lee W. H. Nodal gap structure in the noncentrosymmetric superconductor LaNiC_2_ from magnetic-penetration-depth measurements. New J. Phys. 13, 123022 (2011).

[b27] Rodriguez-Carvajal J. FULLPROF: a program for Rietveld refinement and pattern matching analysis. Abstr. Satell. Meet. Powder Diffr. XV Congr. IUCr 127 (1990).

[b28] Sato T. . Pseudogap of Charge-Density-Wave Compound SmNiC_2_ Studied by High-Resolution Photoemission Spectroscopy. J. Phys. Soc. Jpn. 79, 044707 (2010).

[b29] Matthias B. T., Suhl H. & Corenzwit E. Spin Exchange in Superconductors. Phys. Rev. Lett. 1, 92–94 (1958).

[b30] Arrott A. Criterion for Ferromagnetism from Observations of Magnetic Isotherms. Phys. Rev. 108, 1394–1396 (1957).

[b31] Long Y., Zheng C. Z., Luo J. L., Cheng Z. J. & He Y. S. Heat capacity of the ternary compounds RENiC_2_(RE = Dy, Ho, Er and Y). J. Appl. Phys. 89, 3523–3525 (2001).

[b32] Morales F., Mendivil L. F. & Escamilla R. Chemical pressure in SmNiC_2−x_B_x_ compounds: evidence of a quantum critical behavior. J. Phys.: Condens. Matter 26, 455602 (2014). 25318982 10.1088/0953-8984/26/45/455602

[b33] Stauffer D. & Aharony A. Introduction to percolation theory (CRC Press, 1994)

[b34] Koehler W. C. Magnetic Properties of Rare‐Earth Metals and Alloys. J. Appl. Phys. 36, 1078–1087 (1965).

[b35] Yusuf S. M. . Possible quantum critical point in (La_1−x_Dy_x_)_0.7_Ca_0.3_MnO_3_. Phys. Rev. B 74, 144427 (2006).

