# Supplementary material for: Tuning the ferromagnetic phase in the CDW compound SmNiC2 via chemical alloying
Source: Sci Rep. 2016 May 25;6:26530. doi: 10.1038/srep26530 (PMC4879522; doi:10.1038/srep26530)
Supplement: Supplementary Information [file srep26530-s1.pdf]

Tuning the ferromagnetic phase in the CDW compound $SmNiC_2$ via chemical alloying

G. Prathiba[1], I. Kim[1], S. Shin[1], J. Strychalska[2], T. Klimczuk[2*], T. Park[1*]

[1] *Department of Physics, Sungkyunkwan University, Suwon 440-746, Korea*

[2] *Faculty of Applied Physics and Mathematics, Gdansk University of Technology, Narutowicza 11/12, 80-232 Gdansk, Poland*

*Corresponding authors: Tuson Park (e-mail: tp8701@skku.edu), Tomasz Klimczuk (e-mail: tomasz.klimczuk@pg.edu.pl)

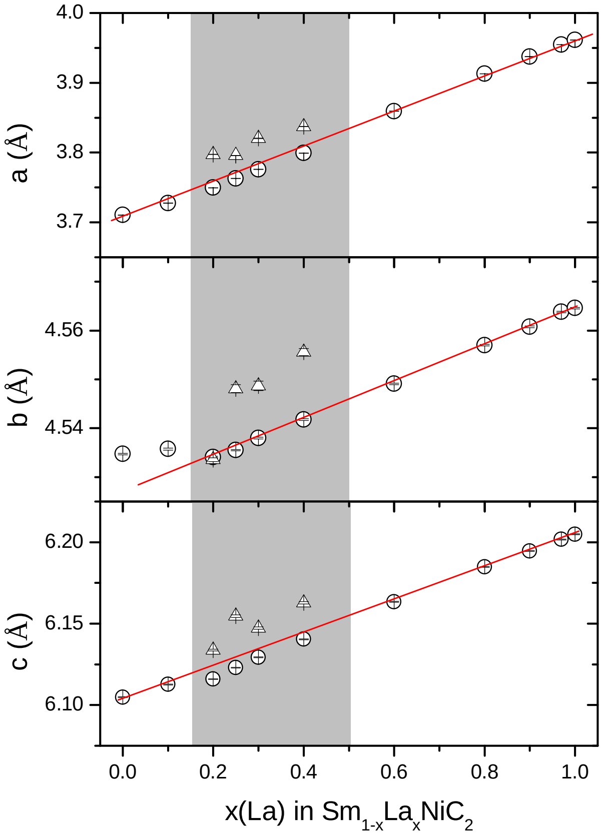

Figure S1. Lattice parameters for $Sm_{1-x}La_xNiC_2$. Solid line is a guide to the eyes and the shaded area represents the La concentration range where the secondary phase is detected (see the main text for details)

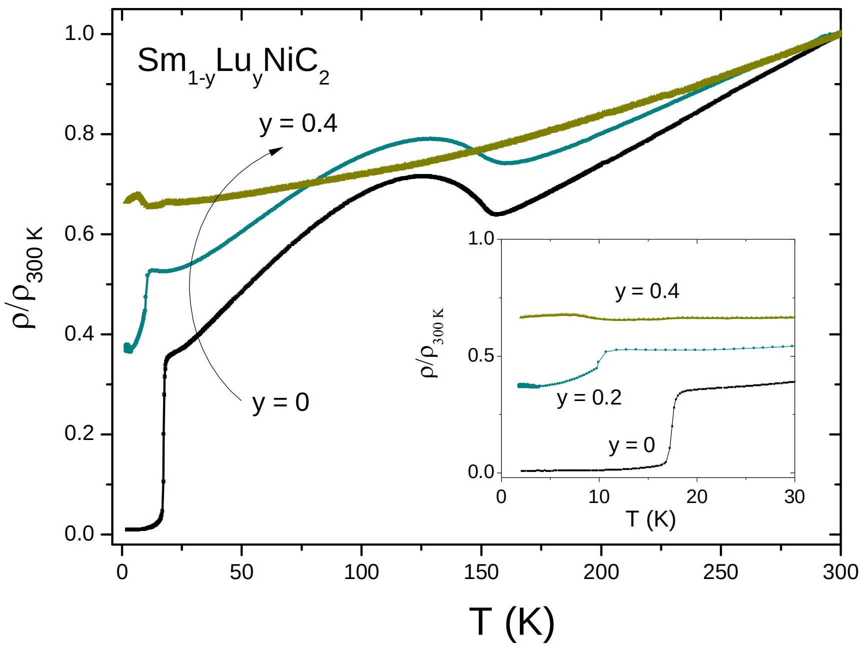

Figure S2. Electrical resistivity for Lu-doped $SmNiC_2$. (a) Temperature dependence of the normalized electrical resistivity for $Sm_{1-y}Lu_yNiC_2$ (y = 0, 0.2, 0.4). (b) Low-temperature resistivity is magnified to show the FM phase transition for y = 0, 0.2, and 0.4.

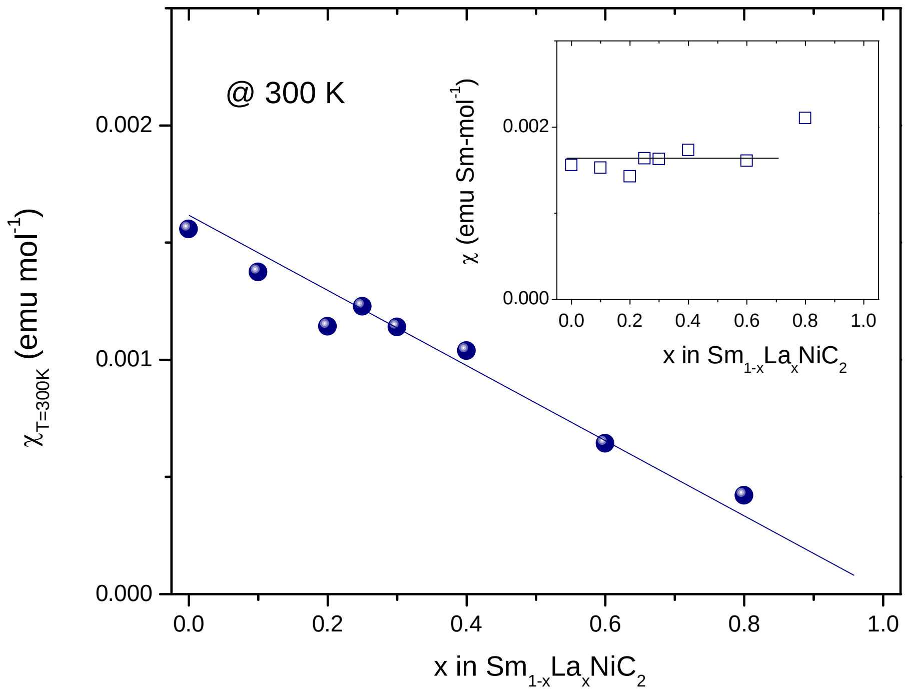

Figure S3. Magnetic susceptibility at 300 K ($\chi_{T=300K}$) is plotted as a function of composition x for $Sm_{1-x}La_xNiC_2$, $0 \le x \le 0.8$. Inset: The magnetic susceptibility divided by the Sm concentration, $\chi_{T=300K}/(1-x)$, is plotted as a function of x. Solid lines are guides to eyes.

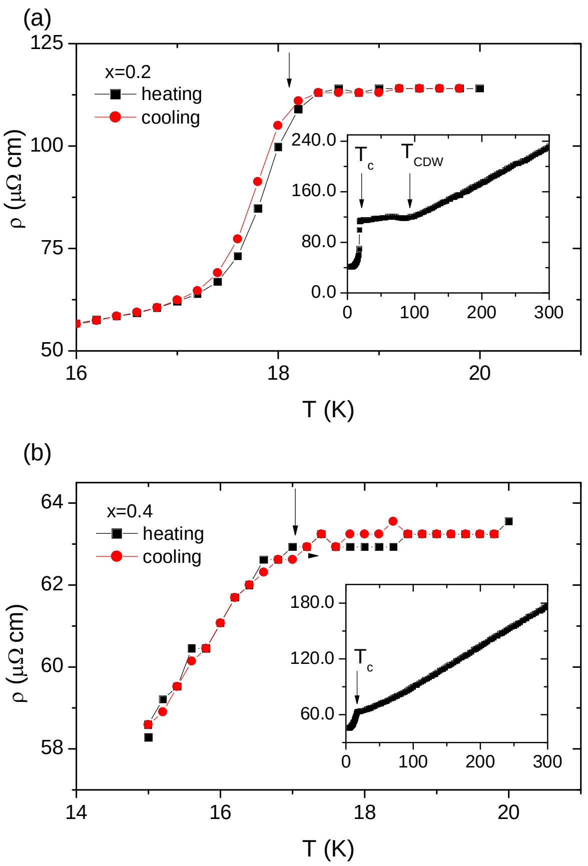

Figure S4. Electrical resistivity of $Sm_{1-x}La_xNiC_2$ is selectively shown for x=0.2 in panel (a) and 0.4 in panel (b). Black squares and red circles represent the resistance taken with increasing and decreasing temperature, respectively. The main panel describes the resistance near the Curie temperature T_C, while the insert describes the resistance over the whole temperature range up to 300 K. We note that a hysteresis loop is observed for x=0.2, while it disappears for x=0.4.
